# Anterolateral thigh free flaps with T-shaped pedicles and multiple venous anastomosis for extremity reconstruction

**DOI:** 10.1097/MD.0000000000026575

**Published:** 2021-07-09

**Authors:** Jun Ho Lee, Hwan Jun Choi, Si Hyun Kwak, Da Woon Lee, Min Sung Tak, Jin Seok Kang

**Affiliations:** a Department of Plastic and Reconstructive Surgery, Soonchunhyang University Cheonan Hospital, Cheonan; b Department of Plastic and Reconstructive Surgery, Soonchunhyang University Seoul Hospital, Yongsan-gu, Seoul, South Korea.

**Keywords:** anastomosis, extremity, flap, free flap, pedicle

## Abstract

The anterolateral thigh free flap is one of the most preferred options for reconstructing soft tissues of the extremities and vascular anastomosis is one of the most important factors for flaps survival. T-anastomosis and double venous anastomosis have been widely used for increasing flap survival. This report shows both application of T-shape pedicle and multiple venous anastomosis to each 43 cases for extremity reconstruction that have not been described so far in the literature and it showed the necessity of multiple anastomosis. The locations of the lesions were 8 upper extremities (4 hands, 3 forearms, and 1 upper arm) and 35 lower extremities (5 forefeet, 6 dorsal feet, 4 plantar feet, 11 ankles, and 9 lower legs). We applied T-shaped arterial pedicle to limited anatomical area that had 2 or more major arterial communication sites to overcome the obstruction by reverse flow from communication vessels when 1 of the 2 anastomosis was obstructed. We classified multiple venous anastomosis according to flow direction and the vascular connections between the superficial and deep veins. In result, 37 cases survived completely but 2 flaps developed severe necrosis (>50%) because of infection and hematoma and 4 flaps developed partial necrosis due to wound infection. In conclusion, T-shaped pedicle and multiple venous anastomosis is a method to improve free flap survival and useful in cases where sacrificing a dominant vessel is inevitable or those in which only 1 vessel remains.

## 1 Introduction

Flaps are an essential tool for soft-tissue reconstruction. Flap complexity ranges from local to free and perforator flaps. Microvascular surgery failure rates have decreased with advances in techniques and instrumentation. The anterolateral thigh (ALT) free flap is a preferred option for reconstructing soft tissues of the extremities because of ease of harvest and low donor-site morbidity with high reliability and versatility. Some studies have demonstrated that the ALT flap can be safely extended to include adjacent vascular territory perfused by a single perforator from the lateral circumflex femoral artery (LCFA).^[1]^ A vascular anastomosis is one of the most important factors for flap survival during microvascular surgery. Anastomosis methods and modifications, such as the T-anastomosis^[2]^ and double venous anastomosis,^[3]^ have been widely used to reconstruct the extremities. But it is unclear whether or not multiple anastomoses reduce the risk of free-flap failure. Some reports demonstrated that performing 2 venous anastomoses reduced incidence of flap failure compared with those of a single vein anastomoses.^[4,5]^ Based on these results, we hypothesized that multiple venous anastomosis is helpful for extremities reconstruction because it reduces the possibility of total occlusion by increasing the number of anastomoses. In this report, we performed ALT free flap with T-shaped pedicles and multiple venous anastomoses for 43 cases of extremity reconstruction and it showed the necessity of multiple anastomoses.

## 2 Patients and methods

We report 43 cases of ALT free flaps with T-shaped pedicles and multiple venous anastomoses to reconstruct extremities and the report period ranged from January 2011 to August 2014. All patients were between 14 and 72 years of age with an average of 46.5 years (11 females and 32 males). The locations of the lesions were 8 upper extremities (4 hands, 3 forearms, and 1 upper arm) and 35 lower extremities (5 forefeet, 6 dorsal feet, 4 plantar feet, 11 ankles, and 9 lower legs). The reasons for the soft tissue defects were diabetic foot ulcers (14 patients) and trauma (29 patients). Wound size ranged from 9.0 cm^2^ (3 × 3 cm^2^) to 242.0 cm^2^ (22 × 11 cm^2^). Institutional Review Board of Soonchunhyang University Cheonan Hospital (Institutional Review Board FILE No.: 2021-03-018) reviewed and discussed the ethical issues in this article. Table 1 summarizes the data and clinical history of patient.

**Table 1:** The data and clinical history of patient.

Gender	Age	Underlying disease (DM, HTN, CRF)	Defect location	Cause	Preopertive angiographic intervention	Postoperative angioraphic result	Complication
M	14	None	Hand, left	Mechanical trauma	X	Intact	Partial necrosis
M	21	None	Forefeet, right	Mechanical trauma	X	Intact	Dehiscence
M	21	None	Forearm, right	Mechanical trauma	X	Intact	None
M	26	None	Plantarfeet, left	Traffic accident	X	Intact	None
M	67	DM	Forefeet, left	Diabetic ulcer	O	Margin blood flow decreased	Partial necrosis
M	23	None	Ankle, left	Mechanical trauma	X	Intact	Dehiscence
M	65	DM, HTN	Dorsalfeet, right	Diabetic ulcer	O	Margin blood flow decreased	Partial necrosis
M	17	None	Hand, left	Traffic accident	X	Intact	None
M	62	DM	Ankle, right	Traffic accident	X	Intact	None
F	48	HTN	Ankle, right	Mechanical trauma	X	Intact	None
F	65	DM, HTN	Lowerleg, left	Diabetic ulcer	O	Intact	None
M	71	DM, HTN, CRF	ankle, right	Traffic accident	X	Margin blood flow decreased	Partial necrosis
M	64	DM, HTN	Ankle, left	Mechanical trauma	X	Intact	None
F	62	HTN	Lowerleg, right	Traffic accident	X	Intact	Dehiscence
F	65	DM, HTN	Ankle, left	Mechanical trauma	X	Intact	Dehiscence
M	24	None	Forearm, right	Mechanical trauma	X	Intact	None
F	20	None	Upperarm, left	Traffic accident	X	Intact	None
F	51	HTN	Plantarfeet, left	Mechanical trauma	X	Intact	None
F	53	DM, HTN, CRF	Dorsalfeet, left	Diabetic ulcer	O	Margin blood flow decreased	Partial necrosis
M	56	HTN	Lowerleg, left	Mechanical trauma	X	Intact	None
M	24	None	Ankle, right	Mechanical trauma	X	Intact	None
M	71	DM, HTN	Forefeet, right	Diabetic ulcer	O	Severe blood flow loss	Necrosis
F	59	DM, HTN, CRF	Ankle, left	Diabetic ulcer	O	Intact	None
M	64	DM, HTN	Forearm, left	Mechanical trauma	X	Intact	None
F	65	DM	Plantarfeet, right	Diabetic ulcer	X	Intact	None
M	37	None	Hand, right	Mechanical trauma	X	Intact	None
M	21	None	Ankle, left	Traffic accident	X	Intact	None
M	72	DM, HTN	Dorsalfeet, left	Diabetic ulcer	O	Intact	None
M	45	None	Lowerleg, right	Traffic accident	X	Intact	None
M	61	DM	Plantarfeet, right	Diabetic ulcer	X	Intact	Partial necrosis
M	56	HTN	Ankle, left	Mechanical trauma	X	Intact	Infection
M	20	None	Hand, right	Mechanical trauma	X	Intact	None
M	40	HTN	Lowerleg, right	Traffic accident	X	Intact	None
M	48	DM, HTN	Ankle, right	Traffic accident	X	Margin blood flow decreased	Partial necrosis
F	67	DM, HTN	Dorsalfeet, right	Diabetic ulcer	O	Severe blood flow loss	Necrosis
F	72	DM, HTN	Lowerleg, right	Diabetic ulcer	O	Margin blood flow decreased	Partial necrosis
M	16	None	Ankle, right	Traffic accident	X	Intact	None
M	35	HTN	Lowerleg, left	Traffic accident	X	Intact	None
M	66	DM, HTN	Dorsalfeet, right	Diabetic ulcer	O	Intact	Infection
M	50	DM, HTN	Dorsalfeet, right	Diabetic ulcer	O	Intact	Infection
M	44	HTN	Forefeet, right	Traffic accident	X	Intact	None
M	60	DM	Lowerleg, left	Diabetic ulcer	X	Intact	Partial necrosis
M	16	None	Lowerleg, left	Traffic accident	X	Intact	None

CRF = chronic renal failure, DM = diabetic mellitus, F = female, HTN = hypertension, M = male.

### 2.1 Surgical technique

**Figure 1:**
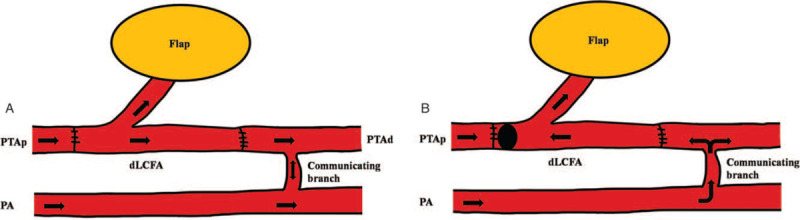
Anatomical limited application of T-shaped arterial pedicle. The PA connects to the PTA in the posterior ankle joint area through a communication branch. A, Arterial flow is in the forward direction when the PTA is used as the recipient vessel and the T-shaped pedicle is interpositioned between each end of the divided PTA without a thrombosis. B, We hypothesized that the PA functions as a back-up vascular system by reverse flow when a proximal anastomosis of the PTA is obstructed. (dLCFA = descending branch of the lateral circumflex femoral artery, PA = peroneal artery, PTAp and PTAd = proximal and distal end of posterior tibial artery, V1p and V1d = proximal and distal end of venae comitant 1 of the recipient, V2p and V2d = proximal and distal end of venae comitant 2 of the recipient, VC1 and VC2 = venae comitantes of the flap).

**Figure 2:**
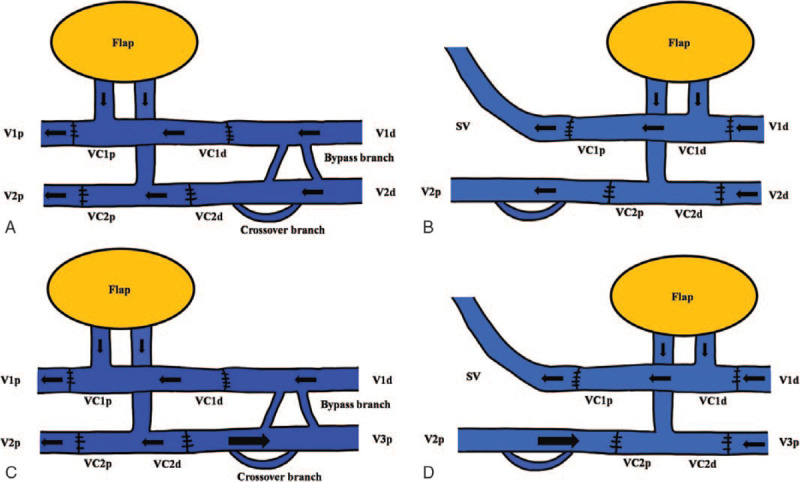
Classification of multiple venous anastomoses according to flow direction and the vascular connection. A, Type I is the classical form in which the venae comitant (VC) of the flap and recipient vessel are anastomosed in the forward flow direction. B, In type II, a VC of the flap was anastomosed to a superficial vein of the recipient which allowed back-up flow when the deep-to-deep anastomosis is obstructed. C, Type III shows a bidirectional anastomosis when recipient and flap vein flow is opposed. Reverse venous blood drainage is possible because of the 2 communicating branches, such as the crossover branch between the 2 VCs and the bypass branch of each vein. D, Type IV is a combined form of types II and III that show a deep to superficial and bidirectional anastomosis. (V1p and V1d = proximal and distal end of the VC1 of the recipient, V2p and V2d = proximal and distal end of VC2 of the recipient, VC1p and VC1d = proximal and distal end of VC1 of the flap, VC2p and VC2d = proximal and distal end of VC2 of the flap, SV = superficial vein).

The patients were examined preoperatively by computed tomography angiography or real-time angiography to confirm dominant vascular flow and validate the existence of any endovascular disease. Hand-held Doppler was applied routinely to confirm and evaluate the recipient vessels and ALT perforators noninvasively. We applied T-shaped arterial pedicle to limited anatomical area that showed 2 or more major arterial communication sites, such as the ankle, foot, finger, or hand region. We hypothesized that arterial flow is maintained by reverse flow of the distal anastomosis due to arterial communication when the proximal anastomosis has trouble. For example, anterior tibial artery, peroneal artery, and posterior tibial artery (PTA) anastomoses are connected by communicating vessels in the ankle region, allowing maintenance of foot perfusion. In the posterior ankle joint area, if PTA is used as a recipient vessel and a T-shaped pedicle is interpositioned between each end of the divided PTA, peroneal artery functions as back-up vascular system by reverse flow when a proximal anastomosis of the PTA is obstructed. This hypothesis involves vessel communication around the anastomosis site as well as characteristics of the T-anastomosis (Fig. 1). For venous anastomoses, the vanae comitantes (VC) of LCFA also has a T-shaped pedicle that includes both ends of the VC and perforating vessel. They are anastomosed to at least 3 venous ends, such as the VC of the recipient site and a superficial vein. We classified multiple venous anastomoses according to flow direction and the vascular connection between the superficial and deep veins. Type I is the classical form in which VC of the flap and recipient vessel are anastomosed in a forward flow direction. A VC of the flap in the type II is anastomosed to a superficial recipient vein, allowing back-up flow when the deep-to-deep anastomosis is obstructed. Type III is a bidirectional anastomosis when recipient and flap vein flow is opposed. Type IV is a combined form of types II and III that shows a deep to superficial and a bidirectional anastomosis (Fig. 2). In operation, the first procedure of anastomoses is selection of T-shape pedicle that has largest diameter perforator and the 2 ends of the descending branch of the LFCA. After insetting the flap, the harvested T-shape pedicle was positioned between each end of the divided recipient vessel, and 2 end-to-end anastomoses were performed. Next, we anastomosed the dominant VC of the flap and recipient with the largest diameter vessel. More than 2 freestyle venous anastomoses were made between the superficial recipient vein and the flap VC or between the recipient VC and the flap's remaining VC based on similar vessel diameter. In process of anastomosis between similar diameters of vessel, the flow of recipient and flap vein might conflict by opposite flow direction in anastomosis site (Types II and III). In this situation, the united flow followed more powerful pressure direction by pressure gradient although the direction of united flow was reverse.

## 3 Results

The flap size ranged from 16 cm^2^ (4 × 4 cm) to 375 cm^2^ (25 × 15 cm), with a median of 66.1 cm^2^. The venous anastomoses were comprised of 13 type I, 14 type II, 9 type III, and 7 type IV. Of the 43 cases, 37 survived completely, and 2 flaps developed severe necrosis (>50%) because of wound infection due to methicillin-resistant *Staphylococcus aureus* infection and a deep tissue hematoma (4.6% severe flap loss rate). These 2 cases healed successfully with a splint-thickness skin graft and a reverse sural island flap. Four flaps also developed partial necrosis due to wound infection but healed after revising the margins under local anesthesia. Flaps needed to be reduced in 12 patients with/without liposuction. We confirmed intact arterial and venous bidirectional flow at the anastomosis sites by postoperative angiographic computed tomography.

### 3.1 Case 1

**Figure 3:**
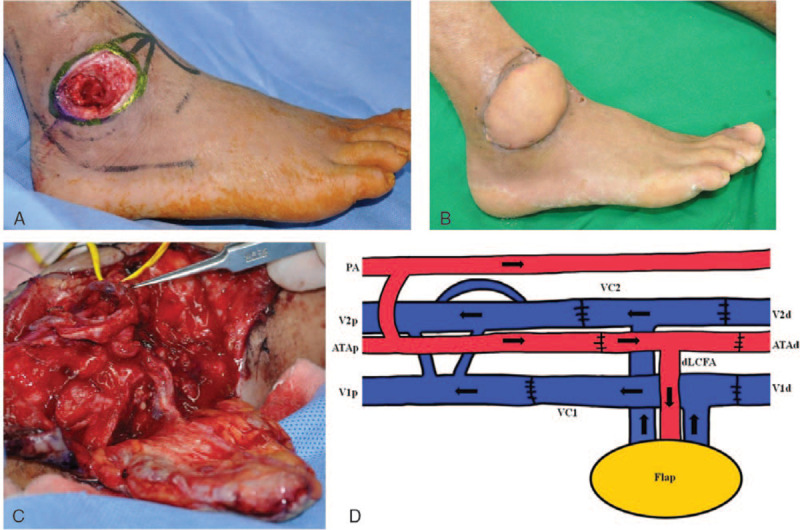
A, A 64-year-old man with chronic bursitis and bony exposure on the right lateral malleolar area. B, The right lateral malleolar area wound was resurfaced with an anterolateral thigh (ALT) free flap. C, T-shaped pedicles with multiple venous anastomoses were used in ALT free flap. D, A diagram of anastomoses. The proximal and distal ends of the dLCFA were anastomosed to the proximal and distal ends of the anterior tibial artery using a T-shape pedicle to preserve recipient flow (connection between PA and ATA was not visible in the operative field). Then, the 2 proximal and distal ends of the 2 VCs of the flap and recipient were anastomosed (type I). (ATAp and ATAd = proximal and distal end of the anterior tibial artery, PA = peroneal artery).

A 64-year-old man had chronic bursitis with bony exposure on the right lateral malleolar area. The wound was reconstructed with an ALT free flap using T-shaped pedicles and multiple venous anastomoses. The proximal and distal ends of the descending branch of the LCFA, including a musculocutaneous perforator and 2 VC, were selected during harvesting. The proximal and distal ends of the descending branch of the LCFA were anastomosed to the proximal and distal ends of the anterior tibial artery using a T-anastomosis to preserve recipient flow. Then, the 2 proximal and distal ends of the 2 VCs of the flap and recipient vessels were anastomosed (Fig. 3).

### 3.2 Case 2

**Figure 4:**
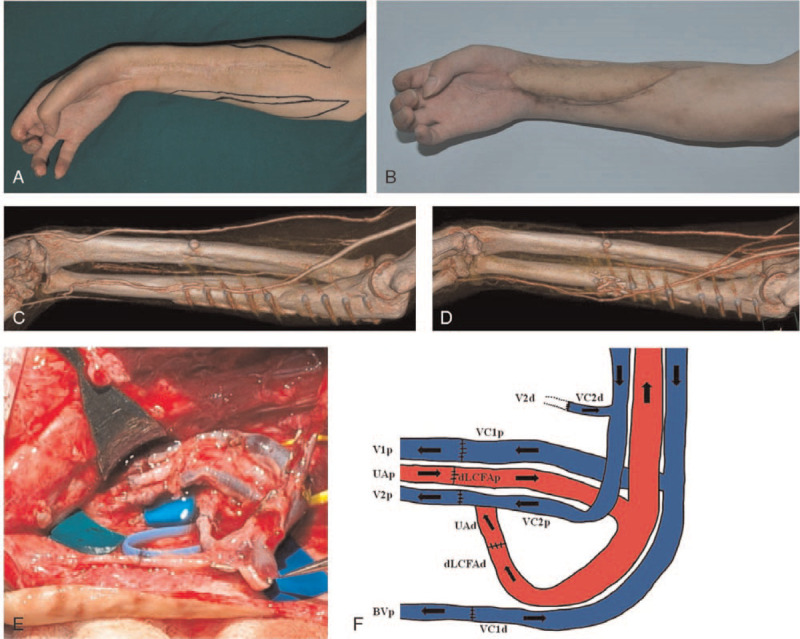
A, A 24-year-old man has a Volkmann's contracture and radial artery occlusion in the right forearm due to a crushing injury. B, The wound was reconstructed with an ALT free flap. C, A radial artery occlusion was detected on a preoperative angiographic computed tomography (CT) scan. D, Patency of ulnar artery was preserved, as shown on a postoperative angiographic CT scan. E, T-shaped pedicles with multiple venous anastomoses were used in ALT free flap. F, A diagram of anastomoses. The proximal and distal ends of the dLCFA were anastomosed to the proximal and distal ends of the ulnar artery. Both ends of VC1 of the flap were anastomosed to the proximal end of V1 and the proximal branch of the basilic vein (BVp). Because the directions of flow in the BVp and VC1d were opposed, united flow would follow a more powerful pressure direction (type IV). Both ends of VC2 of the flap were anastomosed to V2 of the recipient in a forward direction (Anastomosis between V2d and VC2d was invisible in (C)) (dLCFAp and dLCFAd = proximal and distal ends of the descending branch of the lateral circumflex femoral artery, UAp and UAd = proximal and distal ends of the ulnar artery, VC1p and VC1d = proximal and distal end of venae comitant 1 of flap, VC2p and VC2d = proximal and distal end of venae comitant 2 of flap).

A 24-year-old man had Volkmann's contracture and a radial artery occlusion in the right forearm due to a crush injury. The wound was reconstructed with an ALT free flap using T-shaped pedicles and multiple venous anastomoses. The proximal and distal ends of the descending branch of the LCFA were anastomosed to the proximal and distal ends of the ulnar artery using a T-anastomosis to preserve recipient flow. Then, both ends of the 2 VCs of the flap were anastomosed to 2 proximal and 1 distal end of the VCs of the recipient and a superficial vein (basilica vein) (Fig. 4).

### 3.3 Case 3

**Figure 5:**
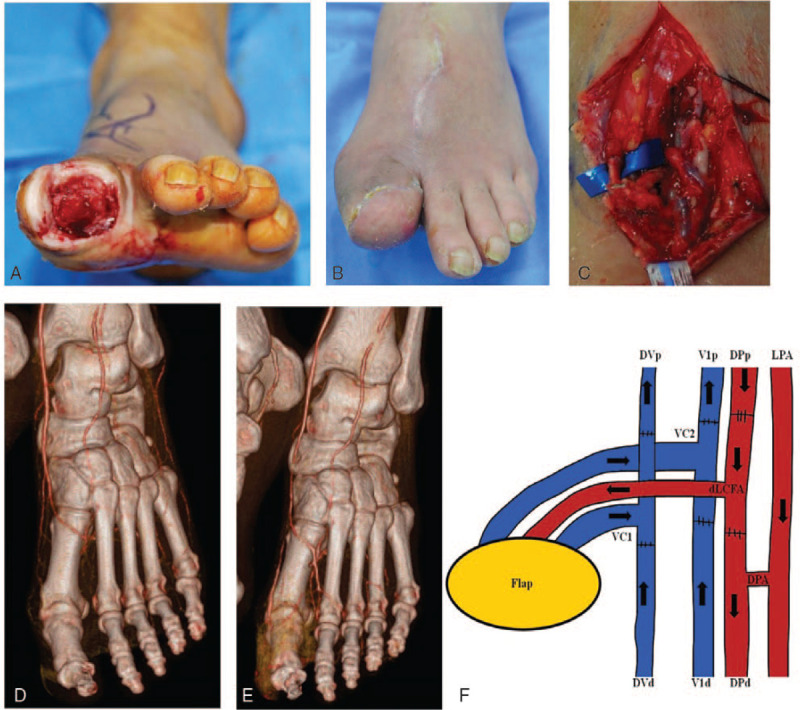
A, A 54-year-old man with a chronic diabetic ulcer and bony exposure on the left greater toe. B, The wound was reconstructed with an ALT free flap. C, Decreased vascular flow in great toe was detected on a preoperative angiographic CT scan. D, Patency of distal flow from anastomosis site was preserved, as shown on a postoperative angiographic CT scan. E, T-shaped pedicles with multiple venous anastomoses were used in ALT free flap. F, Diagram of anastomoses. The proximal and distal ends of the dLCFA were anastomosed to the proximal and distal ends of the DP. The DP was connected to the LPA by the DPA, and this allows a connection between the dorsal and plantar arterial systems (LPA was not visible in the operative field). Both ends of VC1 of the flap were anastomosed to the proximal and distal ends of the DV. Both ends of VC2 of the flap were anastomosed to the proximal and distal ends of V1 (type II). (DPA = deep plantar artery, DPp and DPd = proximal and distal ends of the dorsalis pedis, LPA = lateral plantar artery.).

A 54-year-old man had a chronic diabetic ulcer with bony exposure of the left greater toe. The wound was reconstructed with an ALT free flap. The proximal and distal ends of the descending branch of the LCFA were anastomosed to the proximal and distal ends of the dorsalis pedis to preserve recipient flow. Then, the proximal ends of the 2 VCs of the flap were anastomosed to the proximal and distal ends of the dorsal vein. The distal ends of the 2 VCs of the flap were anastomosed to the proximal and distal ends of the recipient VC (Fig. 5).

## 4 Discussion

For anatomical limited application of T-shaped arterial pedicle, we thought that there was a higher possibility of obstruction using T-shape pedicle that has 2 anastomosis sites rather than simple end-to-end anastomosis. And then, we assumed that when T-shape pedicle located around 2 or more major arterial communication sites and 1 of 2 anastomosis was obstructed, it could overcome the obstruction by reverse flow from communication vessels. This hypothesis involves vessel communication around the anastomosis site and has the advantages of a T-anastomosis. Kim et al^[2]^ clinically applied the T-anastomosis to reconstruct microsurgical free flaps and demonstrated that the T-anastomosis preserves donor and recipient vessels and balances pressure in the flap through normal physiological flow. This method is useful when reconstructing severely injured vessels or in cases of chronic vasculopathy of the extremities because it is important to preserve patency of the recipient vessel for the distal circulation.^[2]^

**Figure 6:**
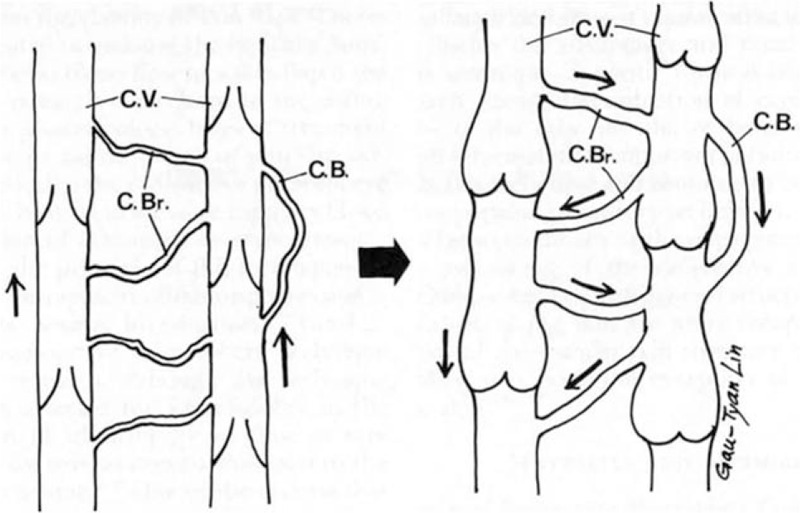
Model of venous drainage in the reverse flow direction. (Left) Venous blood is normally drained in a forward direction. Several bypass branches (C.Br.) between the venae comitantes (C.V.) and the crossover branch (C.B.) in each vein are noted. (Right) These branches enable the venous blood to drain in the reverse direction through crossover and bypass branches. The venae comitantes and branches engorge markedly because of the high venous pressure. (Reprinted with permission from Lin SD, Lai CS, Chiu CC. Venous drainage in the reverse forearm flap. Plast Reconstr Surg. 1984;74(4):508–12).

The multiple venous anastomosis refers connection between superficial and deep veins or bidirectional anastomosis with a T-shape pedicle including both ends of a VC and a perforating vessel. Some studies have demonstrated the roles and efficacy of perforating vein between superficial and deep venous system in a radial forearm free flap. Tahara et al^[6]^ showed that the perforating vein communicates with the radial comitans (deep vein system) and with the cutaneous venous system at the cubital fossa. Both venous drainage systems were restored when the perforating vein was contained in the pedicle. The advantages of this approach are as follows: more than 2 anastomoses provide a fail-safe compared with only 1 vein anastomosis; restores both the deep and cutaneous venous drainage systems; and a variety of veins suitable for the anastomosis are retained.^[6]^ Alan Turner and Smith reported that double separate venous anastomoses for a radial artery forearm flap improve success and minimize morbidity.^[3]^ In that report, the cephalic vein and VC were anastomosed end to side onto the internal jugular vein. The perforating veins were ligated during flap harvest to prevent a thrombosis from spreading between veins.^[3]^ Based on these results, we assumed that a direct anastomosis between the superficial and deep vein systems without a perforating vessel and 2 independent T-shaped anastomoses would have a similar effect compared with that of the aforementioned forearm flap case; thus, it could be applied to a general free flap venous anastomosis. The type II has the aforementioned anastomosis between the deep and superficial veins, which allows back-up flow when the deep-to-deep anastomosis is obstructed by a thrombosis. Bidirectional supercharging means that the united flow follows the more powerful pressure direction and maintains physiological flow, although the direction of united flow is reversed when recipient and flap vein flow conflicts due to the opposite flow direction. This hypothesis is based on Lin's report that reverse drainage of venous blood is possible due to 2 communicating branches: the crossover branch between the 2 VC and a bypass branch of each vein, even though the vein valves are intact (Fig. 6).^[7]^ This hypothesis suggests that a freestyle anastomosis between similar diameter vessels is possible regardless of flow direction. Type III has a bidirectional and independent venous anastomosis including reverse flow and allows back-up flow through a bypass branch when one vessel is obstructed. Based on these hypotheses, we classified multiple venous anastomoses into 4 types. Among the 4 types, it is difficult to say which type is superior for successful flap reconstruction. Ichinose et al^[8]^ reported that dual anastomoses of the separate venous systems (superficial and deep) result in a lower incidence of venous insufficiency than that of a single anastomosis (0.7% vs 7.5%). In contrast, the incidence of venous insufficiency (11.5% vs 7.5%) of dual anastomoses in only the venous system was not different.^[8]^ This result suggests that type II or IV, which includes a separate anastomosis between the superficial and deep veins, is superior with a lower incidence of venous insufficiency.

Whether or not multiple venous anastomoses reduce the risk of free-flap failure is unclear. Various reports suggest performing multiple venous anastomoses to reduce the thrombosis rate during free flap surgery. Ross et al^[4]^ showed that successful flap reconstruction in patients undergoing 2 venous anastomoses was 145 of 147 (98.6%) compared with 323 of 345 (93.6%) patients undergoing a single anastomosis. Ahmadi et al^[5]^ demonstrated that performing 2 venous anastomoses was associated with 36% reduced incidence of flap failure and 34% reduced incidence of venous thrombosis compared with those of a single vein anastomoses based on a meta-analysis. However, other authors have suggested that dual venous drainage may actually reduce venous blood flow velocity, which can cause a thrombosis. Hanasono et al^[9]^ measured blood velocity in each of 2 VCs by Doppler ultrasonography and argued that venous blood velocity is significantly greater after a single venous anastomosis than after either 2 veins or when 2 venous anastomoses are performed. That report also showed that when 1 VC is occluded, blood velocity in the second VC increases significantly. The first result means that performing 2 anastomoses should theoretically increase the thrombosis risk because of the low velocity, which is associated with a thrombosis. However, the second result shows that when 1 VC is occluded, the other has normal flow without thrombosis due to increased blood velocity. We confirmed that these results support the necessity of multiple venous anastomoses. The multiple venous anastomoses is helpful for extremities with chronic vasculopathy, including varicose veins, venous stasis ulcers, or extensive lower extremity wounds that require multiple venous anastomoses because it reduces the possibility of total occlusion by increasing the number of anastomoses.

Thus, we assumed that flap survival rate would increase and demonstrated that vascular anastomosis without arterial insufficiency or venous congestion was possible using T-shaped pedicles and multiple venous anastomoses. T-anastomosis^[2]^ and double venous anastomosis^[3]^ have been widely used to reconstruct the extremities, but this report shows both application of T-shape pedicle and multiple venous anastomosis to each case that have not been described so far in the literature.

However, some limitations exist, such as the anatomical limitation due to the requirement of 2 arterial communication vessels around the anastomosis site and more than 2 VC; more time and effort are required for multiple venous anastomoses; and increased possibility of torsion or kinking of the pedicle due to multiple anastomoses.

A limitation of our report is that we only applied to ALT free flaps; thus, it is difficult to generalize the application to all reconstructed free flaps. The reasons for selecting the ALT free flap vary. First, the anatomy of the descending branch of the LCFA and the multiple perforating branches to the surrounding rectus femoris and vastus lateralis muscles as well as the septocutaneous vessels to the skin could form a T-shaped pedicle easily.^[10]^ The subscapular arterial system has several large diameter branches, such as the branch to the serratus anterior muscle or the circumflex scapular artery that can be used as a T-anastomosis pedicle.^[2]^ Second, an ALT flap can be harvested with a skin paddle as large as that of an abdominal perforator flap and provides a longer pedicle (8–16 cm) with a vessel diameter > 2 mm.^[10]^ Third, the pedicle contains the VC essential for the multiple venous anastomoses. Finally, the descending branch of the LCFA appears to be relatively spared from peripheral vascular disease, hypertension, and diabetes.^[11]^ Thus, the ALT free flap provides the reconstructive surgeon with multiple flap choices based on the versatility of this system. Although we only performed the ALT free flap, it can be applied to other free flap reconstructions when they show the aforementioned characteristics of an ALT free flap. Another limitation is that we hypothesized the concept of back pedicle based on vascular anatomy and physiology, and could not hemodynamically confirm whether or not arterial communication vessels function as back pedicle by reverse flow when 1 of 2 arterial anastomoses was obstructed; united flow followed more powerful pressure direction by pressure gradient in venous anastomoses site.

## 5 Conclusions

T-shaped pedicles with multiple venous anastomoses is a safe and alternative method that improves free flap survival. This is useful in cases where sacrificing a dominant vessel is inevitable or when only 1 vessel remains. It has several advantages, such as preserving recipient flow of a major vessel injury, rebuilding deficient peripheral vascular flow, and dispersing blood flow in small or extended flaps. It can also be applied where many venous anastomoses are required, including varicose veins, venous stasis ulcers, or extensive lower extremity wounds. The T-shaped pedicles with multiple venous anastomoses with a free flap can be used to reconstruct any skin or soft tissue defect where 2 or more major arterial anastomoses are located.

## Author contributions

**Conceptualization:** Dawoon Lee.

**Data curation:** Sihyun Kwak, Dawoon Lee.

**Resources:** Jinseok Kang.

**Writing – original draft:** Junho Lee, Hwan Jun Choi.

**Writing – review & editing:** Sihyun Kwak, Minsung Tak.
